# The utility of sentinel Lymph node biopsy in the lateral neck in papillary thyroid carcinoma

**DOI:** 10.3389/fendo.2022.937870

**Published:** 2022-07-25

**Authors:** Xing-qiang Yan, Zhao-sheng Ma, Zhen-zhen Zhang, Dong Xu, Yang-jun Cai, Zeng-gui Wu, Zhong-qiu Zheng, Bo-jian Xie, Fei-lin Cao

**Affiliations:** ^1^ Department of Surgical Oncology, Taizhou Hospital of Zhejiang Province, Wenzhou Medical University, Linhai, China; ^2^ Department of plastic surgery, Enze Hospital of Taizhou Enze Medical Center (Group), Luqiao, China

**Keywords:** sentinel lymph node biopsy (SLNB), papillary thyroid carcinoma (PTC), lymph node metastases (LNMs), lateral neck dissection, lateral compartment-oriented neck dissection

## Abstract

**Background:**

Regional lymph node metastases (LNMs) are very common in papillary thyroid carcinoma (PTC) and associate with locoregional recurrence. The appropriate management of cervical lymph nodes is very important. Therefore, this study evaluated the application of sentinel lymph node biopsy (SLNB) in the lateral neck in PTC patients.

**Methods:**

This prospective study was conducted from 1 November 2015 to 31 December 2017 and recruited 78 PTC patients treated with SLNB in the lateral neck and prophylactic lateral neck dissection (compartments II–IV) followed by thyroidectomy or lobectomy and central neck dissection.

**Results:**

There were 78 PTC patients enrolled and sentinel lymph nodes (SLNs) were detected among 77 patients. A total of 30 patients were diagnosed with SLN metastases (SLNMs). The remaining 47 patients were pathologically negative of SLN, whereas 4 patients were found with metastases in the non-SLN samples. The detection rate, sensitivity, specificity, and accuracy rate of SLNB in the lateral neck were 98.7%, 87.1%, 98.7%, and 93.6%, respectively. However, the values varied greatly in each specific compartment of the lateral neck, and all of them were no more than 80%. These 34 PTC patients diagnosed with lateral compartment LNM (LLNM) were more likely to be younger (41.38 vs. 48.95 years old, p = 0.002) and exhibit extrathyroidal extension (56.8% vs. 31.7%, p = 0.026) and central compartment LNM (66.7% vs. 12.1%, p < 0.001). Tumors located in the upper third of the thyroid lobe also had a significantly higher probability of LLNM compared with those in middle or inferior location (66.7% vs. 35.3% vs. 34.8%, p = 0.044). At last, age (OR=0.912, p = 0.026), tumor location (upper vs inferior, OR=17.478, p = 0.011), and central compartment LNM (OR=25.364, p < 0.001) were independently predictive of LLNM.

**Conclusions:**

SLNB can help surgeons to identify some PTC patients who may benefit from therapeutic lateral neck dissection and protect some patients from prophylactic lateral neck dissection. However, it cannot accurately indicate specific lateral compartment-oriented neck dissection. Meanwhile, LLNM is more likely to occur in PTC patients with younger age or upper pole tumors or central compartment LNM.

## Introduction

Thyroid cancer is the most common malignant tumor in the endocrine system and head and neck tumors, causing 586,000 cases worldwide and ranking 9th in incidence in 2020 (1). Papillary thyroid carcinoma (PTC) accounts for the vast majority of thyroid cancers. Regional lymph node metastases (LNMs) are very common in patients with PTC, about up to 80% in the central compartment and up to 60% in the lateral compartment of the neck (2–4). Although the incidence of regional LNM is greatly high in PTC, the impact of regional LNM on the prognosis remains unclear. Meanwhile, regional LNM has been reported in association with a higher rate of locoregional recurrence (2, 5, 6). Surgical resection of clinically nodal-positive disease in PTC is considered to improve the results of both recurrence and survival. Therefore, it is generally believed that therapeutic cervical lymph node dissection is indicated in PTC patients with clinically evident cervical LNM. The 2015 American Thyroid Association guidelines recommend that therapeutic lateral neck dissection should be performed for patients with biopsy-proven metastatic lateral cervical lymphadenopathy (7). By contrast, routine prophylactic modified radical neck dissection is not advised and has not been proved with benefit. Therefore, the appropriate management of cervical lymph nodes is very important to PTC patients, which is helpful to improve survival, decrease regional recurrence, and avoid overtreatment.

Sentinel lymph node biopsy (SLNB) is used to assess the status of regional draining lymph nodes, and it has become a standard method for treating several types of human malignant tumors, especially for melanoma and breast cancer (8, 9). However, the application of SLNB in the treatment of PTC has not been thoroughly studied. SLNB in thyroid carcinoma was first reported in 1998 (10). In the last two decades, many studies have evaluated the feasibility and utility of SLNB in PTC patients, and most of these studies focused on the application of SLNB to replace the prophylactic central neck dissection. However, there are few studies that investigated the application of SLNB in lateral compartment lymph nodes in PTC patients. In a previous study, we investigated the utility of SLNB in central compartment lymph nodes and found that this technique was feasible, safe, and useful (11). Therefore, we investigated the application of SLNB in lateral compartment lymph nodes in this study and hypothesized that this technique could identify and remove cervical nodal disease in the primary surgery and eventually avoid a second surgery.

## Patients and methods

### Ethics statement

This study was approved by the Ethics Committee of Taizhou Hospital of Zhejiang Province, and written informed consent was obtained from all the PTC patients prior to enrollment. The collection and analysis of all data were anonymous.

### Patients

This prospective study was conducted on 78 patients with PTC. All patients underwent surgical treatment by SLNB in the lateral compartment lymph nodes and prophylactic lateral neck dissection (compartments II–IV) at our hospital from 1 November 2015 to 31 December 2017. All of the enrolled patients were diagnosed with PTC preoperatively based on fine-needle aspiration biopsy (FNAB) and imaging evaluation of cervical lymph nodes by ultrasound with or without computed tomography before operation. Patients with clinically occult lymph nodes or suspicion of LNM but without confirmation by FNAB were included in this study, while patients diagnosed preoperatively with lateral compartment LNM (LLNM) based on FNAB were excluded. Patients with previous neck surgery were also excluded from this study.

### Surgical procedure

All the surgery operations were performed by experienced surgeons who had performed more than two hundred thyroid surgeries per year within the past decade and had experience with handling thyroid SLN procedures. A transverse low-collar skin incision was followed by separation of the skin flap and a longitudinal incision in the linea alba cervicalis. Then, the thyroid pseudocapsule was carefully opened to completely expose the thyroid gland without injury to the capsule. The primary tumor location was confirmed intraoperatively according to the preoperative ultrasonography, then approximately 0.5 ml of carbon nanoparticle suspension or 1% methylene blue dye was injected into the parenchyma surrounding the primary tumor using a 27-gauge needle. Within minutes, the lateral neck region along the jugular vein was exposed and explored, searching for the stained lymph nodes. The black- or blue-stained lymph nodes were identified *via* tracing the dyeing lymphatic vessels. These stained lymph nodes, defined as sentinel lymph nodes (SLNs), were carefully removed and sent to the pathology department for frozen biopsy and routine pathology. Subsequently, prophylactic lateral neck dissection (compartments II–IV) was performed, followed by total thyroidectomy or lobectomy and central neck dissection. The remaining nonstained lymph nodes in the lateral compartment of neck, defined as non-SLNs, were sent for routine pathology. All the diagnoses were made by two experienced pathologists, and all the final diagnoses were based on routine pathology findings.

### Data analysis

Descriptive statistics were used to analyze the characteristics of the patients and tumors. Univariate analysis and multivariate logistic regression analysis were applied to analyze the association between the LNM and clinicopathological factors. The t-test and chi-square test were used for the measurements and count data, respectively. SPSS version 19 (SPSS Inc., Chicago, IL, USA) was used in all of the statistical analyses, and statistical significance was defined as a p-value less than 0.05.

## Results

The characteristics of all patients and tumors are shown in **Table 1**. In total, 78 patients with low to intermediate risk PTC were recruited in this study. There were 63 (80.8%) women and 15 (19.2%) men with a mean age of 45.65 years old (ranging from 21 to 71 years old). A total of 42 (53.8%) patients were diagnosed with PTC, while 36 (46.2%) patients were diagnosed with papillary thyroid microcarcinoma (PTMC). A total of 18 (23.1%) patients showed cervical adenopathy by the preoperative imaging evaluation. Nearly half of the patients presented extrathyroidal extension (37, 47.4%), and one-third of the patients were diagnosed with multifocality (31, 39.7%) and thyroiditis (26, 33.3%). Majority of the patients presented BRAF mutations (84.6%). About two-fifths of the patients (35, 44.9%) had transient hypoparathyroidism in post-operation, but all of them recovered to normal in six months. Nearly one-third of the patients (27, 34.6%) received adjuvant radioactive iodine therapy after surgery. In the final pathology findings, 49 (62.8%) patients were diagnosed with LNM, of whom 45 (57.7%) patients had central compartment LNM and 34 (43.6%) patients had LLNM. The stained lymph nodes were identified among 77 patients, and the remaining one patient without dyeing lymph node was pathologically negative of lymph nodes in the study. A total of 2,019 lymph nodes were removed, of which 473 SLN and 881 non-SLN were removed from the lateral compartment of the neck. All the patients were followed up every 6 months for the first two years and then annually thereafter. The mean follow-up time was 62 months, ranging from 47 to 71 months. No one relapsed during the follow-up.

**Table 1:** Patient demographics and tumor characteristics (n = 78).

Characteristic	Value
Sex	
Male	15 (19.2%)
Female	63 (80.8%)
Age (years old)	
Mean ± SD (range)	45.65 ± 11.09 (21-71)
Tumor location	
Left	31 (39.7%)
Right	47 (60.3%)
Upper	21 (26.9%)
Middle	34 (43.6%)
Inferior	23 (29.5%)
Cervical adenopathy	
Present	18 (23.1%)
Absent	60 (76.9%)
Tumor type	
PTMC	36 (46.2%)
PTC	42 (53.8%)
Multifocality	
Multifocal	31 (39.7%)
Unifocal	47 (60.3%)
Extrathyroidal extension	
Present	37 (47.4%)
Absent	41 (52.6%)
Thyroiditis	
Present	26 (33.3%)
Absent	52 (66.7%)
BRAF mutation	
Present	66 (84.6%)
Absent	12 (15.4%)
Hypoparathyroidism	
Present	35 (44.9%)
Absent	45 (55.1%)
RAI therapy	
Present	27 (34.6%)
Absent	51 (65.4%)
LNM in total	49 (62.8%)
Central compartment	45 (57.7%)
Lateral compartment	34 (43.6%)
Compartment II	19 (24.4%)
Compartment III	26 (33.3%)
Compartment IV	20 (25.6%)
SLN in lateral compartment	77 (98.7%)
Compartment II	58 (74.4%)
Compartment III	62 (79.5%)
Compartment IV	58 (74.4%)
Number of nodes	
SLN (mean)	473 (6.1)
Non-SLN (mean)	881 (11.3)
Total-LN (mean)	2019 (25.9)
Follow-up time (range) (month)	62.4 (47-71)

PTMC, papillary thyroid microcarcinoma; PTC, papillary thyroid carcinoma; LNM, lymph node metastases; SLN, sentinel lymph node; Total-LN, total lymph nodes.

The results of SLNB are shown in **Table 2**. A total of 77 patients succeeded in SLNB in the lateral compartment of the neck. There were 30 patients who were diagnosed with SLN metastases (SLNM), of whom 20 were detected with further metastases in the non-SLN samples. The remaining 47 patients were pathologically negative of SLN, whereas 4 patients exhibited metastases in the non-SLN samples. The detection rate, sensitivity, specificity, and accuracy rate of SLNB in the lateral compartment of the neck were 98.7%, 87.1%, 98.7%, and 93.6%, respectively. However, the values varied greatly in the different areas of the lateral compartment of the neck. There were 58 patients who were detected to have SLN in compartment II, of whom 11 patients were SLN positive and 47 patients were SLN negative. However, 3 patients exhibited metastases in the non-SLN samples in the group of SLN negative. The detection rate, sensitivity, specificity, and accuracy rate were 74.4%, 58.4%, 74.4%, and 70.5%, respectively. A total of 62 patients showed SLN in compartment III, of whom 19 patients were positive in SLN and 3 patients were positive in non-SLN among the remaining patients. The detection rate, sensitivity, specificity, and accuracy rate of compartment III were 79.5%, 68.6%, 79.5%, and 75.6%, respectively. In compartment IV, SLN was found among 58 patients, of whom 10 patients were SLN positive. Meanwhile, 4 patients were pathologically positive in non-SLN among the patients with SLN negative. The detection rate, sensitivity, specificity, and accuracy rate of compartment IV were 74.4%, 53.1%, 74.4%, and 69.2%, respectively.

**Table 2:** Results of SLNB in the lateral compartment (n = 78).

	Lateral compartment	Compartment II	Compartment III	Compartment IV
SLN detection	77	58	62	58
SLN positive	30	11	19	10
SLN negative	47	47	43	48
Misdiagnosis	4	3	3	4
Missed diagnosis	1	20	16	20
Detection rate	98.7%	74.4%	79.5%	74.4%
Sensitivity	87.1%	58.4%	68.6%	53.1%
Specificity	98.7%	74.4%	79.5%	74.4%
Accuracy rate	93.6%	70.5%	75.6%	69.2%

SLNB, sentinel lymph node biopsy; SLN, sentinel lymph node.

The association between LLNM and clinical factors was also analyzed. PTC patients with LLNM were significantly younger than those without LLNM (41.38 vs. 48.95 years old, p = 0.002). Tumors located in the upper third of the thyroid lobe had a significantly higher probability of LLNM compared with those in the middle or inferior location (66.7% vs. 35.3% vs. 34.8%, p = 0.044). Patients with extrathyroidal extension were more likely to have LLNM than those without extrathyroidal extension (56.8% vs. 31.7%, p = 0.026). What is more, PTC patients with central compartment LNM indicated a significantly higher incidence of LLNM (66.7% vs. 12.1%, p < 0.001). However, other factors such as sex, cervical adenopathy, thyroiditis, multifocality, tumor size, and BRAF mutations did not show statistical significance for LLNM (**Table 3**). A multivariate logistic regression analysis was performed to determine whether these factors were independently correlated with LLNM. At last, age (OR=0.912, p = 0.026), tumor location (upper vs inferior, OR=17.478, p = 0.011), and central compartment LNM (OR=25.364, p < 0.001) were independently predictive of LLNM (**Table 4**).

**Table 3:** Comparison of patients with and without LNM in the lateral compartment (n = 78).

	Metastases (n=34)	Non-metastases (n=44)	P-value
Sex			
Female/Male	27/7	36/8	0.789
Age (mean ± SD) (years old)	41.38 ± 11.00	48.95 ± 10.08	0.002*
Tumor location			
Left/Right	14/20	17/27	0.820
Upper/Middle/Inferior	14/12/8	7/22/15	0.044*
Cervical adenopathy			
Present/Absent	9/25	9/35	0.532
Tumor type			
PTC/PTMC	20/14	22/22	0.438
Multifocality			
Multifocal/Unifocal	16/18	15/29	0.246
Extrathyroidal extension			
Present/Absent	21/13	16/28	0.026*
Thyroiditis			
Present/Absent	12/22	14/30	0.747
BRAF mutation			
Present/Absent	28/6	38/6	0.626
Central compartment LNM			
Present/Absent	30/4	15/29	0.000*

PTMC, papillary thyroid microcarcinoma; PTC, papillary thyroid carcinoma; LNM, lymph node metastases; * p<0.05.

**Table 4:** Multivariate analysis of the clinicopathological factors for patients with lateral compartment LNM (n = 78).

Factors	Odds Ratio	95% CI	P-value
Sex (Female vs. Male)	1.387	0.211 – 9.127	0.734
Age	0.912	0.841 - 0.989	0.026*
Tumor location (Left vs. Right)	1.038	0.254 – 4.248	0.959
Tumor location (Upper vs. Inferior)	17.478	1.924 - 158.785	0.011*
Tumor location (Middle vs. Inferior)	1.930	0.313 - 11.908	0.479
Cervical adenopathy (Present vs. Absent)	0.432	0.060 – 3.108	0.405
Tumor type (PTC vs. PTMC)	2.086	0.519 – 8.383	0.300
Multifocality (Multifocal vs. Unifocal)	3.331	0.639 – 17.371	0.153
Extrathyroidal extension (Present vs. Absent)	0.319	0.082 – 1.244	0.100
Thyroiditis (Present vs. Absent)	0.492	0.089 – 2.721	0.416
BRAF mutation (Present vs. Absent)	0.158	0.018 – 1.368	0.094
Central compartment LNMs (Present vs. Absent)	25.364	4.486 – 143.416	0.000*

PTMC, papillary thyroid microcarcinoma; PTC, papillary thyroid carcinoma; LNM, lymph node metastases; CI, confidence interval; * p<0.05.

## Discussion

Nowadays, the management of cervical lymph node dissection in the treatment of PTC is still one of the most controversial issues. As everyone knows, LLNM in PTC patients is very common, and most of these LNMs may be occult diseases (4). Traditionally, regional LNM has been thought to be associated with a higher rate of locoregional recurrence and decreased survival (2, 5, 6, 12–14). Therapeutic neck dissection, which is advocated for PTC patients with positive nodes by the FNAB, can decrease the recurrence and improve the survival. Although micro-metastases do not carry the same clinical significance as those of macro-metastases, PTC patients with these LNMs will also take a high risk of recurrence and secondary surgery (2). Moreover, there is still a lack of sufficient evidence regarding the confirmation of the efficacy and benefit of prophylactic neck dissection for PTC patients. Due to the efficacy of SLNB for the melanoma and breast cancer, the role of SLNB for PTC patients has been studied. Our previous study investigated SLNB in the central compartment of the neck and suggested that this technique was helpful in the decision-making in central neck dissection for some patients (11). Therefore, we further investigated the role of SLNB in the lateral compartment of the neck and hypothesized that SLNB could identify LNM and could be used as an indicator for selective lateral neck dissection.

SLN serves as the first station in the lymphatic drainage basin, receiving lymph flow from the primary tumor and reflecting the status of the remaining lymph nodes. A successful SLNB requires accurately identifying and localizing the SLN. Generally, SLNB is performed by the use of vital dye, lymphoscintigraphy, or the combined technique. Recently, a review and a meta-analysis both found that the radioisotope technique had a slightly higher SLN detection rate than that of the dye method and the combined technique in PTC patients. However, the sensitivity, specificity, accuracy, and false negative were similar among these three techniques (15, 16). Although the radioisotope technique has advantages in the detection rate, it is very difficult to implement in small hospitals because of the strict management system on radioactive substances. In addition, radiocontamination may occur by accident. The dye method seems to be simple and easy to implement, but a relatively steep learning curve is needed for the surgeons (17). The surgeons need to know the dosage and flow rate of the tracer and protect the lymphatic vessels from destruction during the operation. The combined technique is more complex and difficult to implement, and the outcome is not better than that of other methods. Thus, the vital dye method is by far the most widely used in SLNB worldwide. But the sample size of PTC patients with the radioisotope technique is also increasing (16). In the present study, the dye method was used due to the previous extensive experience of SLNB in the central compartment of the neck.

As we all know, because of the considerable heterogeneity of the SLNB detection rate and its high false-negative rate, the accuracy of this procedure in PTC still remains questionable. There are three studies designed for evaluating the utility of SLNB in the lateral compartment lymph nodes (18–20). The detection rate ranges from 91.8% to 100%, and the accuracy rate ranges from 91.8% to 96.5% in these two studies that used the dye method (18, 20). Another study used the radioisotope technique and showed that the detection rate is 63.8%. The accuracy rate is not provided because the routine lateral neck dissection is not performed (19). In the present study, the detection rate(98.7%) and the accuracy rate(96.3%) of SLNB in the lateral compartment were similar to these previous studies. These results suggested that SLNB might be useful for a decision to perform selective lateral neck dissection in PTC patients. In another word, if any one of the SLNs was positive, the lateral neck dissection should be performed. On the contrary, when all of the SLNs were negative, the lateral neck dissection could be abandoned. In this way, SLN-positive patients may benefit from therapeutic lateral neck dissection, and SLN-negative patients will spare the prophylactic procedure. Meanwhile, the detection rate, sensitivity, specificity, and accuracy rate of each specific lateral compartment of the neck were analyzed in this study. Among these compartments, the detection rate ranged from 74.4% to 79.5%, the sensitivity ranged from 53.1% to 68.6%, the specificity ranged from 74.4% to 79.5%, and the accuracy rate ranged from 69.2% to 70.5%. Due to the relatively low detection rate and accuracy rate in every specific lateral compartment of the neck, lateral compartment-oriented neck dissection could not be performed based on the SLN status in this study. Therefore, SLNB can identify some patients who may benefit from therapeutical lateral neck dissection and protect some patients from prophylactic lateral neck dissection, but SLNB should be abandoned as an index of compartment-oriented neck dissection.

There is a generally accepted assumption that cervical LNM in PTC follows the gradual progression from the central to lateral compartment of the neck. Moreover, lymph nodes that skip metastases to the lateral compartment of the neck are present only in a small number of PTC patients (21, 22). In the present study, we found 45 (57.7%) patients who had central compartment LNM, of whom 30 were accompanied with LLNM simultaneously, and just only 4 (5.1%) patients had skip metastases. Both univariate analysis and multivariate regression analysis showed that LNM in the lateral compartment of the neck was significantly correlated with that in the central compartment of the neck. The details of the relationship between central compartment LNM and LLNM are shown in the supplementary table (**Table S1**). Meanwhile, LLNM was more likely to occur in younger patients in the present study, and a comprehensive analysis in two national databases reported that the outcome of survival was associated with cervical LNM among patients younger than 45 years old (14). Extrathyroidal extension was significantly associated with LLNM in univariate analysis but not in multivariate regression analysis. In the previous study, we found that extrathyroidal extension was independently predictive of LNM in the central compartment of the neck (11). Central compartment LNM might cover the impact of the extrathyroidal extension in multivariate regression analysis in this study. Both univariate analysis and multivariate regression analysis showed that upper pole PTC had a significantly higher probability of LLNM, so that the first station of LNM may be in compartment II rather than the central compartment of the neck among these patients (4). Therefore, factors such as younger age, upper pole tumors, and central compartment LNM may be very important for the decision-making in lateral neck dissection.

However, the present study has several limitations. Firstly, we did not evaluate the role of SLNB in compartment V, which may impact the detection rate and the accuracy rate in this study, even though the LNM in compartment V is relatively rare compared to that in other lateral compartments of the neck, and the vast majority of such metastases are observed only in the context of multicompartment metastases (4, 18, 23). Secondly, the number of patients enrolled in this study is not enough, which might affect the accurate evaluation of the SLNB utility. Thirdly, the mean time of follow-up is about 5 years, which may be not enough for an indolent carcinoma. Since PTC may relapse in 5 years or later after initial surgery, long-term follow-up is necessary to show the reality of recurrence. Fourthly, lateral neck dissection may not be an appropriate method for patients with negative SLN, which may cause unnecessary complications. Active surveillance and long-term follow-up for these patients may be more appropriate.

## Conclusion

In conclusion, SLNB can help surgeons to identify some PTC patients who may benefit from therapeutic lateral neck dissection and protect some patients from prophylactic lateral neck dissection. However, because of the relatively low detection rate and accuracy rate in every specific lateral compartment of the neck, SLNB cannot accurately indicate specific lateral compartment-oriented neck dissection. Meanwhile, LLNM is more likely in PTC patients with younger age or upper pole tumors or central compartment LNM.

## Data availability statement

The raw data supporting the conclusions of this article will be made available by the authors, without undue reservation.

## Ethics statement

The studies involving human participants were reviewed and approved by the Ethics Committee of Taizhou Hospital of Zhejiang Province. The patients/participants provided their written informed consent to participate in this study.

## Author contributions

Study conception and design: F-LC and X-QY. Acquisition of data: X-QY, Z-SM, Z-ZZ, Z-GW, Z-QZ and Y-JC. Analysis and interpretation of data: B-JX, F-LC, DX and X-QY. Drafting of manuscript: X-QY, Z-SM and Z-ZZ. Critical revision of manuscript: B-JX, F-LC and DX. X-QY, Z-SM and Z-ZZ have contributed equally to this work and share first authorship. B-JX and F-LC have contributed equally to this work and share corresponding authorship. All authors contributed to the article and approved the submitted version.

## Funding

This work was kindly supported by the grant from Science and Technology Plan Projects of Taizhou (NO.20ywb33).

## Acknowledgments

The authors would like to thank Prof. Mei-xian Zhang for her valuable suggestion that has helped improve the quality of the manuscript.

## Conflict of interest

The authors declare that the research was conducted in the absence of any commercial or financial relationships that could be construed as a potential conflict of interest.

## Publisher’s note

All claims expressed in this article are solely those of the authors and do not necessarily represent those of their affiliated organizations, or those of the publisher, the editors and the reviewers. Any product that may be evaluated in this article, or claim that may be made by its manufacturer, is not guaranteed or endorsed by the publisher.

## Supplementary material

Supplementary Table 1 The relationship of between CLNM and LLNM.

## References

[1] Sung H Ferlay J Siegel RL Laversanne M Soerjomataram I Jemal A. Global cancer statistics 2020: GLOBOCAN estimates of incidence and mortality worldwide for 36 cancers in 185 countries. CA: Cancer J Clin (2021) 71(3):209–49. doi: 10.3322/caac.21660 33538338

[2] Randolph GW Duh QY Heller KS LiVolsi VA Mandel SJ Steward DL. The prognostic significance of nodal metastases from papillary thyroid carcinoma can be stratified based on the size and number of metastatic lymph nodes, as well as the presence of extranodal extension. Thyroid (2012) 22(11):1144–52. doi: 10.1089/thy.2012.0043 23083442

[3] Thompson AM Turner RM Hayen A Aniss A Jalaty S Learoyd DL. A preoperative nomogram for the prediction of ipsilateral central compartment lymph node metastases in papillary thyroid cancer. Thyroid (2014) 24(4):675–82. doi: 10.1089/thy.2013.0224 PMC3993080 24083952

[4] Cracchiolo JR Wong RJ. Management of the lateral neck in well differentiated thyroid cancer. Eur J Surg Oncol J Eur Soc Surg Oncol Br Assoc Surg Oncol (2018) 44(3):332–7. doi: 10.1016/j.ejso.2017.06.004 PMC8565354 28687430

[5] Pereira JA Jimeno J Miquel J Iglesias M Munné A Sancho JJ. Nodal yield, morbidity, and recurrence after central neck dissection for papillary thyroid carcinoma. Surgery (2005) 138(6):1095–100. doi: 10.1016/j.surg.2005.09.013 16360396

[6] Leboulleux S Rubino C Baudin E Caillou B Hartl DM Bidart JM. Prognostic factors for persistent or recurrent disease of papillary thyroid carcinoma with neck lymph node metastases and/or tumor extension beyond the thyroid capsule at initial diagnosis. J Clin Endocrinol Metab (2005) 90(10):5723–9. doi: 10.1210/jc.2005-0285 16030160

[7] Haugen BR Alexander EK Bible KC Doherty GM Mandel SJ Nikiforov YE. 2015 American Thyroid association management guidelines for adult patients with thyroid nodules and differentiated thyroid cancer: The American thyroid association guidelines task force on thyroid nodules and differentiated thyroid cancer. Thyroid (2016) 26(1):1–133. doi: 10.1089/thy.2015.0020 26462967 PMC4739132

[8] Balch CM Soong SJ Atkins MB Buzaid AC Cascinelli N Coit DG. An evidence-based staging system for cutaneous melanoma. CA: Cancer J Clin (2004) 54(3):131–49. doi: 10.3322/canjclin.54.3.131 15195788

[9] Lyman GH Giuliano AE Somerfield MR Benson AB 3rd Bodurka DC Burstein HJ. American Society of clinical oncology guideline recommendations for sentinel lymph node biopsy in early-stage breast cancer. J Clin Oncol Off J Am Soc Clin Oncol (2005) 23(30):7703–20. doi: 10.1200/jco.2005.08.001 16157938

[10] Kelemen PR Van Herle AJ Giuliano AE. Sentinel lymphadenectomy in thyroid malignant neoplasms. Arch Surg (Chicago Ill 1960) (1998) 133(3):288–92. doi: 10.1001/archsurg.133.3.288 9517742

[11] Yan X Zeng R Ma Z Chen C Chen E Zhang X. The utility of sentinel lymph node biopsy in papillary thyroid carcinoma with occult lymph nodes. PLoS One (2015) 10(6):e0129304. doi: 10.1371/journal.pone.0129304 26046782 PMC4457868

[12] Zaydfudim V Feurer ID Griffin MR Phay JE. The impact of lymph node involvement on survival in patients with papillary and follicular thyroid carcinoma. Surgery (2008) 144(6):1070–7. doi: 10.1016/j.surg.2008.08.034 19041020

[13] Podnos YD Smith D Wagman LD Ellenhorn JD. The implication of lymph node metastasis on survival in patients with well-differentiated thyroid cancer. Am Surgeon (2005) 71(9):731–4. doi: 10.1177/000313480507100907 16468507

[14] Adam MA Pura J Goffredo P Dinan MA Reed SD Scheri RP. Presence and number of lymph node metastases are associated with compromised survival for patients younger than age 45 years with papillary thyroid cancer. J Clin Oncol Off J Am Soc Clin Oncol (2015) 33(21):2370–5. doi: 10.1200/jco.2014.59.8391 26077238

[15] Garau LM Rubello D Morganti R Boni G Volterrani D Colletti PM. Sentinel lymph node biopsy in small papillary thyroid cancer: A meta-analysis. Clin Nucl Med (2019) 44(2):107–18. doi: 10.1097/rlu.0000000000002378 30418209

[16] Garau LM Rubello D Muccioli S Boni G Volterrani D Manca G. The sentinel lymph node biopsy technique in papillary thyroid carcinoma: The issue of false-negative findings. Eur J Surg Oncol J Eur Soc Surg Oncol Br Assoc Surg Oncol (2020) 46(6):967–75. doi: 10.1016/j.ejso.2020.02.007 32098735

[17] Garau LM Rubello D Ferretti A Boni G Volterrani D Manca G. Sentinel lymph node biopsy in small papillary thyroid cancer. A review on novel surgical techniques. Endocrine (2018) 62(2):340–50. doi: 10.1007/s12020-018-1658-5 29968226

[18] Huang N Ma B Guan Q Wang Y Zhou L Wei W. Lateral cervical lymph node mapping in papillary thyroid carcinoma: A prospective cohort study. Chin J Clin Oncol (2018) 45(20):1053–6. doi: 10.3969/j.issn.1000-8179.2018.20.675

[19] Lee SK Kim SH Hur SM Choe JH Kim JH Kim JS. The efficacy of lateral neck sentinel lymph node biopsy in papillary thyroid carcinoma. World J surg (2011) 35(12):2675–82. doi: 10.1007/s00268-011-1254-9 21993615

[20] Markovic I Goran M Buta M Stojiljkovic D Zegarac M Milovanovic Z. Sentinel lymph node biopsy in clinically node negative patients with papillary thyroid carcinoma. J BUON (2020) 25(1):376–82. 32277657

[21] Lee YS Shin SC Lim YS Lee JC Wang SG Son SM. Tumor location-dependent skip lateral cervical lymph node metastasis in papillary thyroid cancer. Head Neck (2014) 36(6):887–91. doi: 10.1002/hed.23391 23733708

[22] Roh JL Park JY Rha KS Park CI. Is central neck dissection necessary for the treatment of lateral cervical nodal recurrence of papillary thyroid carcinoma? Head Neck (2007) 29(10):901–6. doi: 10.1002/hed.20606 17405173

[23] Cabrera RN Chone CT Zantut-Wittmann D Matos P Ferreira DM Pereira PSG. Value of sentinel lymph node biopsy in papillary thyroid cancer: initial results of a prospective trial. Eur Arch Oto-Rhino-Laryngology (2015) 272(4):971–9. doi: 10.1007/s00405-014-3018-2 24695942

