# Supplementary material for: The utility of sentinel Lymph node biopsy in the lateral neck in papillary thyroid carcinoma
Source: Front Endocrinol (Lausanne). 2022 Jul 25;13:937870. doi: 10.3389/fendo.2022.937870 (PMC9357979; doi:10.3389/fendo.2022.937870)
Supplement: Supplementary Table 1 — The relationship of between CLNM and LLNM. [file Table_1.docx]

**Table S1.** The relationship of between CLNM and LLNM.

| No. of CLNM (patients) | 0 (n=33) | 1 (n=15) | 2 (n=6) | 3 (n=6) | ≥4 (n=18) |
| --- | --- | --- | --- | --- | --- |
| No. of patients with LLNM | 4 | 6 | 4 | 5 | 15 |
| No. of patients in each compartment | II (2), III (2) | II (2), III (5), IV (3) | II (2), III (3) | II (3), III (5), IV (4) | II (10), III (11), IV (10) |

CLNM = central compartment lymph node metastasis; LLNM = lateral compartment lymph node metastasis;
